# Escherichia Coli Vertebral Osteomyelitis: A Case Report

**DOI:** 10.7759/cureus.36286

**Published:** 2023-03-17

**Authors:** Ameena Syed, Roshan Afshan, Gantuya Tserenpil, Nouraldeen Manasrah, Gina M Chippi, Abu Fazal Shaik Mohammed

**Affiliations:** 1 Internal Medicine, Detroit Medical Center/Wayne State University/Sinai Grace Hospital, Detroit, USA; 2 Internal Medicine, Weiss Memorial Hospital, Chicago, USA; 3 Medical School, Wayne State University, Detroit, USA

**Keywords:** escherichia coli, pyogenic osteomyelitis, spondylodiskitis, vertebral osteomyelitis, spondylodiscitis

## Abstract

Diagnosis of spondylodiscitis is often challenging, delayed, or even missed due to the uncommonness of the disease, and it can lead to devastating consequences. Therefore, a high index of suspicion is needed for prompt diagnosis and improved long-term outcomes. Vertebral osteomyelitis, or spondylodiscitis, is a rare disease with increasing prevalence due to advanced spinal surgical procedures, nosocomial bacteremia, increased life expectancy, and intravenous drug use. Hematogenous infection is the most common cause of spondylodiscitis. We report a case of a 63-year-old man with a history of liver cirrhosis who initially presented due to abdominal distension. During his hospital stay, he complained of uncontrolled back pain due to *Escherichia coli* spondylodiscitis.

## Introduction

Vertebral osteomyelitis is one of the medical challenges that clinicians face on a routine basis, especially in the early stages of the disease. Unfortunately, it can lead to significant morbidity and mortality in its late stages [1]. Therefore, systematic clinical workup and application of treatment algorithms are necessary to optimize clinical outcomes for a better quality of care. Vertebral osteomyelitis (spondylodiscitis or spinal osteomyelitis) accounts for approximately 3-5% of all cases of osteomyelitis annually [1]. The estimated incidence of vertebral osteomyelitis in the United States is 4.8 per 100,000 and has been increasing lately [1]. Globally, the incidence of vertebral osteomyelitis is suspected to be 1-7 per 100,000 cases [1,2]. 

The most common pathogens causing osteomyelitis vary by the age of the patients. *Staphylococcus aureus* is the most prevalent etiology of acute and chronic hematogenous osteomyelitis in children and adults [3,4]. Osteomyelitis due to contiguous spread or direct inoculation is usually polymicrobial or monomicrobial, while hematogenous osteomyelitis is primarily monomicrobial [4]. Gram-negative organisms are less implicated as a cause of vertebral osteomyelitis [5]. We present a case of *Escherichia coli *as a cause of vertebral osteomyelitis in an elderly male. Risk factors include advanced age, immunosuppression, diabetes mellitus, long-term corticosteroid use, malignancy, malnutrition, untreated prostatitis, intravenous drug use, and, more recently, increasing neurosurgical procedures [6]. The lumbar spine is the most involved, and cervical and thoracic involvement is less common [7]. 

The clinical presentation is nonspecific; thus, delayed diagnosis of up to several months is typical. The most common presenting symptoms include back pain localized to the infected area, and fever (present in only 35-60% of cases) [7]. Therefore, early clinical recognition, even with slight suspicion, is indispensable in reducing morbidity and mortality associated with the condition.

## Case presentation

A 63-year-old male with a medical history of liver cirrhosis, hepatitis C, hypothyroidism, and esophageal varices presented to the emergency department (ED) complaining of shortness of breath at rest. He complained of dizziness when standing up, progressive abdominal distension, swelling in bilateral lower extremities, and worsening chronic back pain. He was also experiencing increased urinary frequency and urgency without fever or dysuria. He is an alcoholic with a remote history of intravenous (IV) drug abuse. He denied having pets, a history of travel, and exposure to animals. On presentation, he was afebrile, with a blood pressure level of 90/66, heart rate of 77 bpm, respiratory rate of 24, and saturating at 96% on room air. A physical exam revealed a normally developed and well-nourished man who appeared in mild distress. He had 2+ pitting edema in bilateral lower extremities and a soft distended abdomen. 

The patients' lab workup revealed elevated BUN, creatinine, liver enzymes, bilirubin, WBC, D dimer, B-type natriuretic peptide (BNP) as well as low platelets. The lab results are depicted in Table 1.

**Table 1:** Patient labs on admission.

Lab workup results	Value	Reference
Sodium	130 mEq/l	136-146 mEq/L
Creatinine	3.53 mg/dl	0.6-1.2 mg/dl
Blood urea nitrogen (BUN)	69 mg/dl	7-18mg/ dl
Bilirubin	5.4 mg/dl	0.1-1.0mg/ dl
Alanine aminotransferase	49 U/L	10-40 U/L
Aspartate aminotransferase	61 U/L	12-38 U/L
Alkaline phosphatase	277 U/L	25-100 U/L
Albumin	2.6g/dl	3.5-5.5g/dl
B-type natriuretic peptide (BNP)	261 pg/ml	<100 Pg/ml
White blood cells (WBC)	18,103/mm3	4500-11,000/mm3
Hemoglobin	12.8gm/dl	13.3-17.1gm/dl
Platelet count	39,000/mm3	150,000-400,000/mm3
D-dimer	31 ng/ml	<250ng/ mL

The chest X-ray, EKG, and urine analysis were unremarkable. A CT scan of the abdomen without contrast revealed cirrhosis with trace perihepatic ascites, splenomegaly, and features suggestive of portal hypertension. A CT scan for pulmonary embolism was negative. Blood cultures grew pan-sensitive *E. coli* in 4/4 bottles, as depicted in Table 2. He was started on intravenous fluids, ceftriaxone, metronidazole, and pain medications. *E. coli* bacteremia resolved on subsequent blood cultures with improving leukocytosis. 

**Table 2:** Antimicrobial susceptibilities for E. coli

S.No	Escherichia coli	Blood cultures	Microbiological susceptibilities
	Antibiotic	Minimum Inhibitory Concentration (MIC)	INTERPRETATION
1	Trimethoprim/Sulfamethoxazole	<=0.5/9.5	Sensitive
2	Tobramycin	<=2	Sensitive
3	Piperacillin/tazobactam	<=2/4	Sensitive
4	Meropenem	<=0.5	Sensitive
5	Gentamicin	<=2	Sensitive
6	Ertapenem	<=0.25	Sensitive
7	Ceftriaxone	<=1	Sensitive
8	Ceftazidime	<=2	Sensitive
9	Cefoxitin	<=4	Sensitive
10	Cefepime	<=1	Sensitive
11	Cefazolin	<=1	Sensitive
12	Aztreonam	<=2	Sensitive
13	Ampicillin	<=4	Sensitive
14	Amp/Sulbactam	<=1/0.5	Sensitive
15	Anti microbial	Culture: E.Coli both bottles	Sensitive

**Figure 1:**
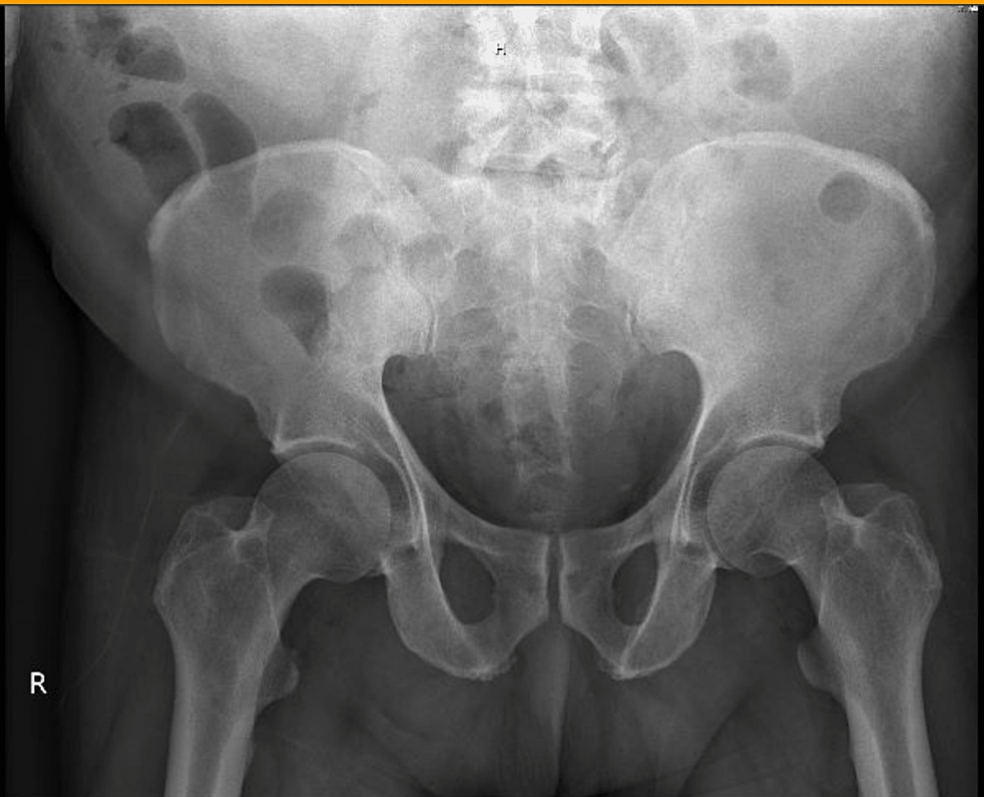
X-ray of pelvis and bilateral hips reveals normal findings

The patient complained of unresolved low back pain (not responding to pain medications) during his hospital stay. However, X-rays of the pelvis and bilateral hips were normal, as depicted in Figure 1.

**Figure 2:**
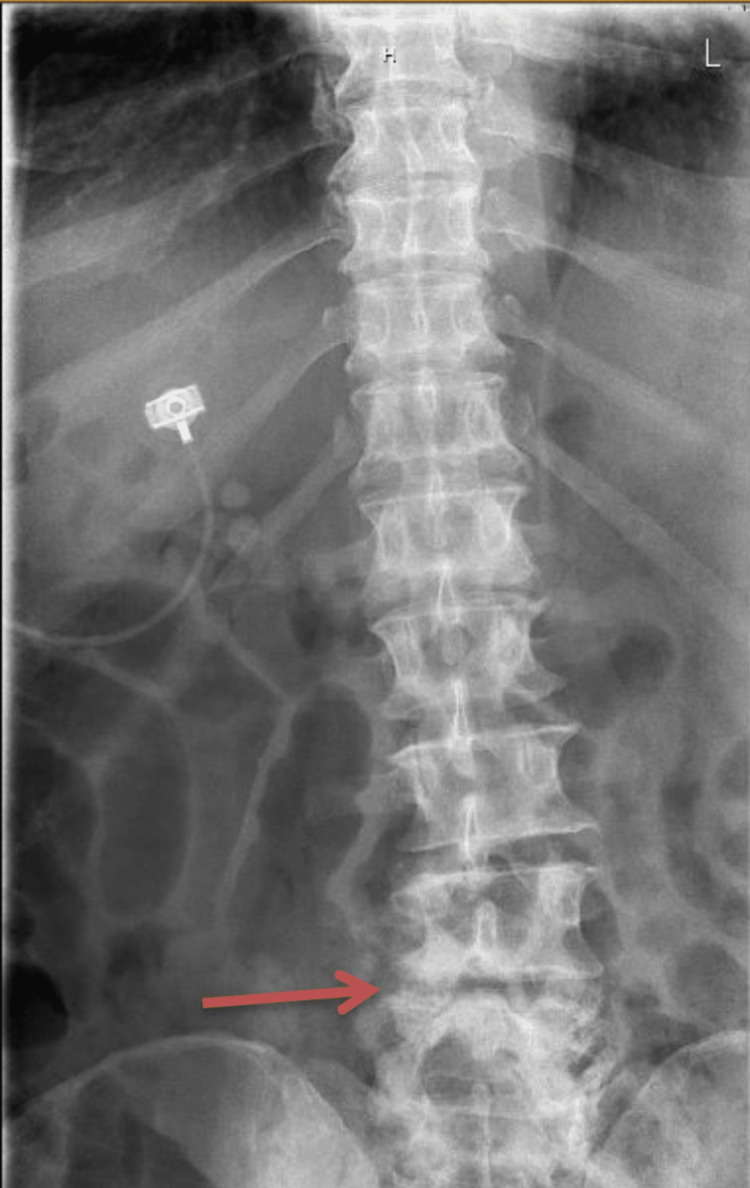
X-ray of the lumbar spine (anteroposterior view): posterolisthesis of L4-L5 (red arrow).

**Figure 3:**
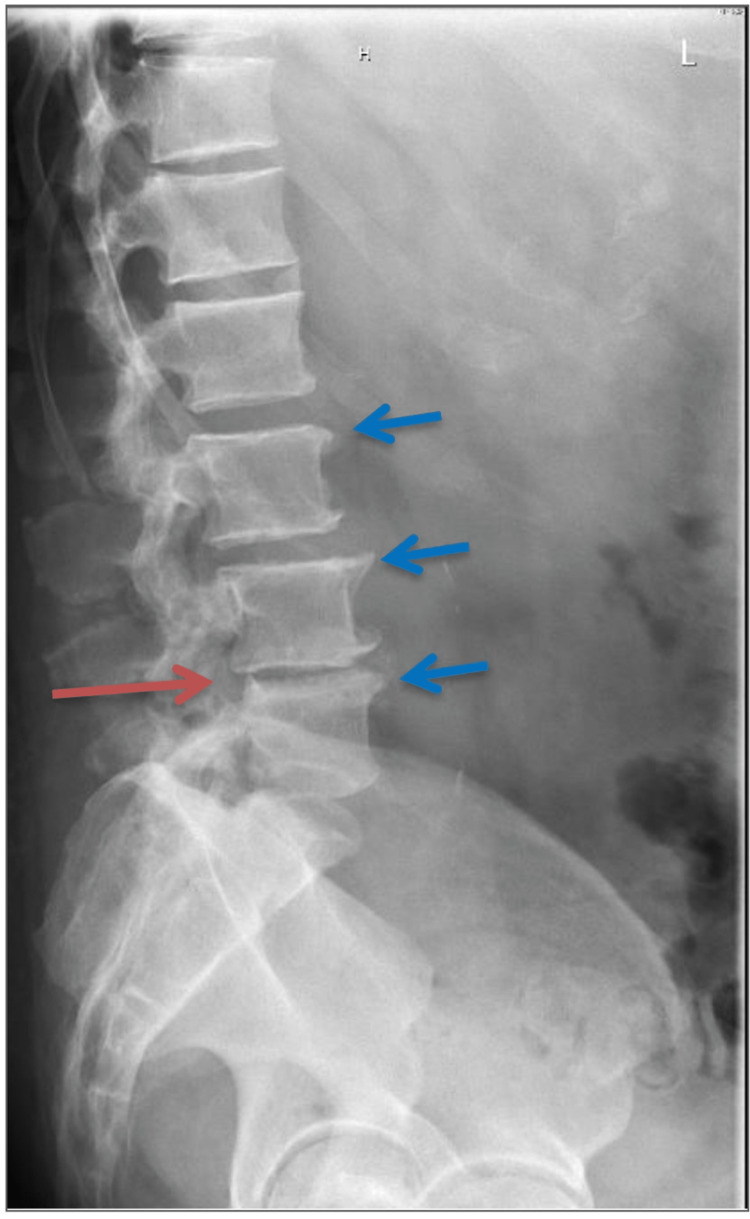
X-ray of the lumbar spine (lateral view) showing the anterior spurs in L3-L5 (blue arrows) and the posterolisthesis of L4-L5 (red arrow).

X-rays of the lumbar spine (LS) showed mild posterolisthesis of L4-5 and narrowing of the first, third, and fourth disc spaces with anterior spurs throughout the lumbar spine, as shown in Figures 2-3.

The initial plan was to discharge the patient to subacute rehabilitation as we considered his lumbago to be chronic. However, due to his intensified low back pain since admission and a prior history of chronic hepatitis C related to IV drug use, we had high suspicion of vertebral osteomyelitis. We then proceeded to perform an MRI with gadolinium contrast of the lumbar spine. We also broadened the antimicrobial coverage by adding vancomycin to existing antibiotics (ceftriaxone and metronidazole). Inflammatory markers were elevated with an erythrocyte sedimentation rate of (ESR) 86mm/h and C-reactive protein (CRP) of 26.5 mg/L. 

**Figure 4:**
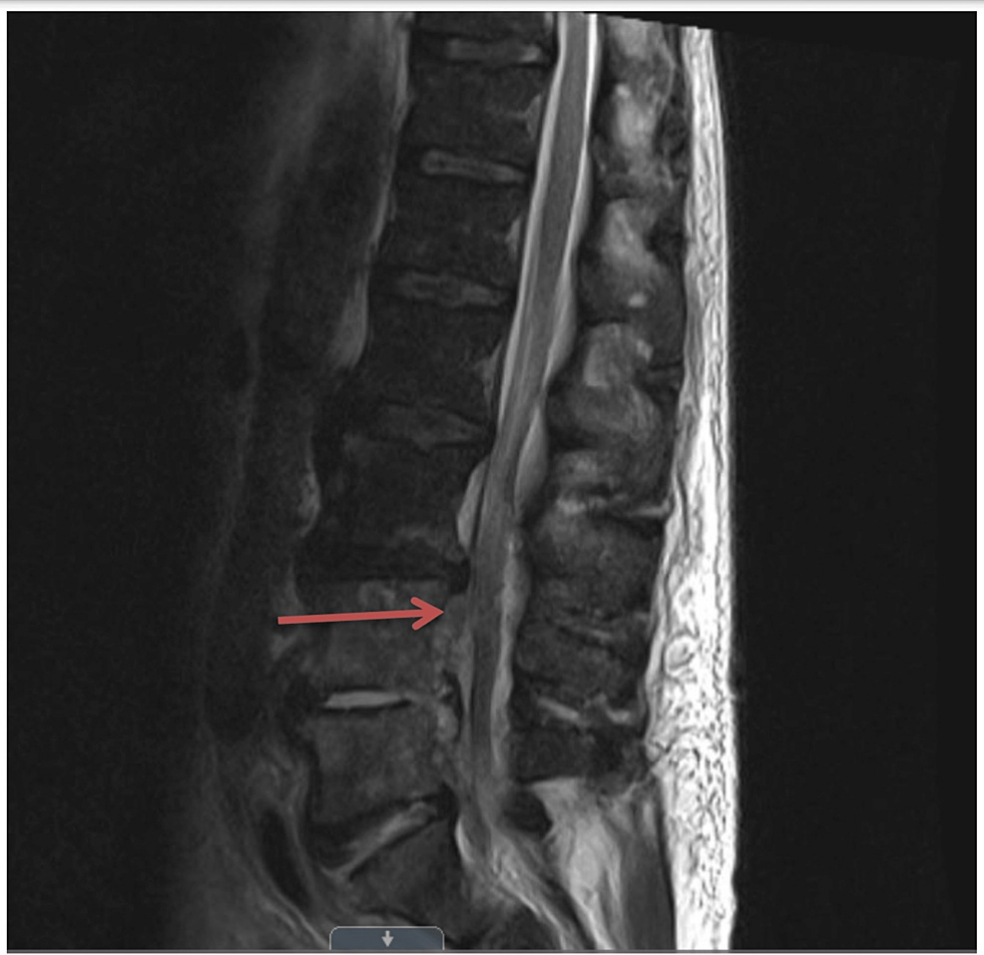
MRI of the lumbar spine with gadolinium contrast shows significant discitis at L4-L5 and L5-S1 with epidural phlegm causing considerable mass effect on the thecal sac (red arrow).

MRI of the LS (Figure 4) with contrast revealed osteomyelitis from L4-S1, with a phlegmon with mass effect on the thecal sac and a possible abscess in the right psoas muscle.

The patient did not exhibit any neurological deficits. Neurosurgery recommended no surgical intervention and recommended an outpatient follow-up in two weeks or sooner in the case of any neurological compromise. They advised fine needle aspiration (FNA) by intervention radiology (IR). CT-guided bone biopsy of the superior endplate of L5 and the L4-L5 disc by IR was performed. As the right psoas abscess was small, it was not considered manageable to drain. The bone biopsy did not reveal any organisms (tissue, fungal, and mycobacterial cultures were negative) but exhibited inflammatory changes. The biopsy results could have been negative due to the early use of antibiotics for *E. coli *bacteremia. We suspected the etiology could be likely the hematogenous spread of *E. coli*. 

At this point, the infectious diseases recommended discontinuing vancomycin. A transthoracic echocardiogram disclosed no evidence of valvular vegetation. The patient deferred subacute rehab as he wished to be discharged home with home health care. The plan was to continue IV ceftriaxone two g/day for eight weeks and follow up with primary care. The patient did not follow up with his primary care physician; however, he had a subsequent visit to ED in three months. During this visit, his ESR was 19mm/h, and CRP was 3.8 mg/L, as depicted in Table 3. He could ambulate without any assistance and had no neurological deficits with a complete resumption of daily activities. 

**Table 3:** Serum markers on follow-up visit

Serum Markers	Inpatient	3 months follow up	Reference
Erythrocyte sedimentation rate	86mm/h	19mm/h	1-13mm/h for males
C-reactive protein	26.5mg/L	3.8mg/L	8-10 mg/L

## Discussion

Vertebral osteomyelitis is a rare spine infection with the presentation of low back pain, fever, or neurologic deficits [1]. Through this case report, we review Gram-negative bacteremia (genitourinary and gastrointestinal sources) as a cause of osteomyelitis. In our case, we did not find a clear source of bacteremia. However, given the history of dysuria, age, and gender of the patient, we think the patient could have had prostatitis. Untreated genitourinary infections can lead to *E. coli* bacteremia, seeding to distant sites and causing vertebral osteomyelitis via the Batson venous plexus [6,8]. MRI is the preferred imaging modality of choice in a patient with suspected osteomyelitis, as it can be missed on CT [2]. In most cases, surgical intervention is commonly needed to drain abscesses and debride sites of infection. 

Prolonged antibiotic therapy is the mainstay in the treatment of vertebral osteomyelitis. Culture and sensitivity results should guide antibiotic therapy. It is acceptable to start empiric broad-spectrum antibiotics while awaiting cultures. Once culture and sensitivity results become available, the antibiotic therapy should be narrowed to targeted coverage. The most commonly used antibiotic regimen is broad-spectrum coverage against Gram-positive and -negative microorganisms, including MRSA with vancomycin plus a third-generation cephalosporin [1]. 

The recommended duration of treatment for osteomyelitis in adults is four to six weeks of parenteral antibiotic therapy to achieve acceptable cure rates with a decreased risk of recurrence [7,9,10]. In cases where the infected bone is completely debrided or amputated, with clean, disease-free margins, a shorter duration of antibiotic therapy is acceptable. Postoperatively, a two-week course of antibiotics is sufficient to treat any residual tissue infection and wound healing of the surgical site [1]. 

Both surgical and medical conservative therapy is proposed for patients with vertebral osteomyelitis. Patients without bony destruction of the endplates can be managed with noninvasive medical intervention. However, patients with vertebral osteomyelitis with neurological symptoms require surgical treatment. A study suggested that surgical intervention has an excellent rate of improving the quality of life compared to conservative therapy for patients with vertebral osteomyelitis who have neurological symptoms [11]. If the patient requires surgical intervention, a less invasive technique, such as an endoscopic approach, is more efficacious than an open approach associated with significant complications [12]. The clinical outcomes for patients with vertebral osteomyelitis are guarded. Even those who recover have residual mild to moderate functional and neurological deficits [2]. 

## Conclusions

In a patient with severe back pain, we recommend that clinicians consider the possibility of vertebral osteomyelitis in their differentials. Vertebral osteomyelitis may develop after hematogenous spread from a distant source. Diagnosis gaps can lead to poor patient outcomes and complications such as bony destruction, abscess formation, neurologic deficits, and even death. Therefore, an immediate diagnosis is warranted to prevent dreaded complications. Imaging with an MRI of the spine is the preferred modality. A multidisciplinary approach involving medical and surgical teams is needed for optimal management. Treatment involves long-term antibiotics.
